# Lymphoma total lesion glycolysis leads to hyperlactatemia and reduction of brain glucose utilization

**DOI:** 10.1038/s41598-022-16562-z

**Published:** 2022-07-25

**Authors:** Hyun Kyung Yi, Jang Yoo, Seok Jin Kim, Joon Young Choi, Kyung-Han Lee

**Affiliations:** 1 Department of Nuclear Medicine, Samsung Medical Center, Sungkyunkwan University School of Medicine, 50 Irwon-dong, Gangnam-gu, Seoul, 16351 Republic of Korea; 2 Department of Nuclear Medicine, Veterans Health Service Medical Center, Seoul, Republic of Korea; 3 Division of Hematology-Oncology, Department of Medicine, Samsung Medical Center, Sungkyunkwan University School of Medicine, Seoul, Republic of Korea

**Keywords:** Cancer, Neurology, Oncology

## Abstract

Clarifying the mechanism of lymphoma-associated hyperlactatemia could help identify patients at risk. Here, 129 non-Hodgkin’s lymphoma patients suspected of blood lactate elevation underwent blood measurement and ^18^F-fluoro-2-deoxyglucose (FDG) positron emission tomography (PET) on the same day. Blood lactate elevation was mild (1.0–2.5 mmol/L) in 60, moderate (2.5–4.0 mmol/L) in 46, and severe (≥ 4.0 mmol/L) in 23 subjects. Subjects with severe lactate elevation had higher lymphoma stage, worse IPI risk, poorer ECOG performance, and higher tumor TLG. Furthermore, there was a linear correlation between blood lactate concentration and lymphoma TLG (Spearman’s r = 0.367; *P* < 0.0001). Brain FDG uptake was low (SUVave < 4.0) in 81 patients that were older, had greater stage and IPI risk, worse ECOG performance, and higher blood lactate. Brain SUVave showed inverse correlation with blood lactate (Spearman’s r = − 0.564; *P* < 0.0001) and lymphoma TLG (Spearman’s r = − 0.252; *P* = 0.0066), as well as with stage, ECOG score, and IPI risk. Multivariable regression analysis confirmed increased blood lactate and lymphoma TLG as significant explanatory variables for reduced brain SUVave (both *P* < 0.0001). Hence, blood lactate elevation in lymphoma patients is the result of glycolytic tumor burden. Since brain cells prefer lactate over glucose as energy source when blood lactate level is increased, this causes proportional reductions of brain FDG uptake. FDG PET/CT can therefore identify high glycolytic lymphoma burden at risk of hyperlactatemia and may provide estimates of its severity by reductions in brain uptake.

## Introduction

Blood lactate level is used in clinical practice as a surrogate for illness severity and to gauge response to therapeutic interventions. In patients with lymphoma, elevation of blood lactate can lead to lactic acidosis, a severe metabolic complication that carries a poor prognosis^1–3^. Significant elevations of circulating lactate itself can have hemodynamic consequences, with higher blood levels and longer time to normalization being associated with greater health risk^4^. Clinicians should therefore be alert for the possibility of hyperlactatemia in lymphoma patients so that appropriate treatment including chemotherapy can be promptly initiated to improve outcomes^5^. However, the diagnosis of lymphoma-induced hyperlactatemia is often delayed, largely due to a lack of readily identifiable symptoms and signs that allow early recognition^5–7^.

A clearer understanding of the precise mechanism of lymphoma-associated hyperlactatemia could help identify patients at risk who require blood testing. It may also help to optimize ways to prevent worsening of comorbidities. Of the two types, type-A lactic acidosis occurs from insufficient oxygen delivery by hypoperfusion or hypoxia that results in anaerobic tissue glycolysis. Examples include septic or cardiogenic shock, and regional ischemia. Type-B lactic acidosis is unassociated with tissue hypoxia or hypoperfusion and is attributed to reduced mitochondrial oxidative phosphorylation without deficient oxygen. Examples include malignancy, liver disease, and certain medications or intoxications^1^. Hyperlactatemia in lymphoma patients is rarely caused by tissue hypoxemia but rather appears related to lactate overproduction by glycolytic tumor^7^. This occurs from lymphoma cells favoring energy production through conversion of glucose to lactate even in the presence of sufficient oxygen^8,9^. Indeed, there is a suspected link between elevated blood lactate and large lymphoma burden^7,10^. Moreover, recent evidence indicates that lactate is more than merely a metabolic waste and has new roles in the tumor microenvironment as a metabolic fuel and signaling molecule^11^.

Glycolytic tumor burden can be measured noninvasively using positron emission tomography (PET) with ^18^F-fluoro-2-deoxyglucose (FDG), a radiolabeled analogue that is taken up in proportion to the glucose utilization rate. Patients with lymphomas are routinely evaluated at diagnosis with FDG PET to assess disease extent^12^. Thus, analysis of FDG PET findings could provide an opportunity to clarify the precise contribution of glycolytic tumor burden to blood lactate level in lymphoma patients, but this has not been previously investigated.

FDG PET further allows three-dimensional mapping of regional glucose metabolism in the brain^13^. This is of significant interest because there are reports of low brain FDG uptake on PET studies of lymphoma patients with hyperlactatemia^11,14^. Whereas glucose has long been established as the obligatory neuronal fuel, it is now recognized that lactate can serve as a major energy substrate for the brain under both hypoglycemic and euglycemic conditions^15–17^. Lactate readily crosses the blood–brain barrier and can cause cerebral glucose-sparing effects in a concentration-dependent fashion^18^. This suggests the possibility that reduced brain FDG uptake on PET might serve as an imaging biomarker that can help identify the presence and assess the severity of hyperlactatemia in lymphoma patients.

In this study, we evaluated lymphoma patients at initial staging or re-staging who underwent blood tests for suspected blood lactate elevation. The contribution of glycolytic tumor burden on blood lactate level was investigated by FDG PET/CT images acquired on the same day of blood testing. We further explored whether reduced brain FDG uptake may serve as an imaging biomarker that correlates with the severity of blood lactate elevation.

## Results

### Clinical characteristics of the entire population and blood lactate groups

The clinical characteristics of the 129 study subjects are summarized in Table 1. The total population had a mean age of 57.9 ± 31.4 y, with 77 males and 52 females. The Eastern Cooperative Oncology Group (ECOG) performance score was ≥ 2 in 58 patients (45%), and the IPI score-based risk was high (4–5) in 61 patients (47%). Ann Arbor stage was I–III in 27 patients and IV in 102 patients; and 31 patients had liver involvement. Mean blood glucose was 109.8 ± 31.4 mg/dL and average blood lactate dehydrogenase (LDH) was 2035 ± 2411 U/L. Blood LDH was increased over the normal range (> 480 U/L) in all subjects except seven cases, who also had blood lactate levels under 2.8 mmol/L.

**Table 1:** Clinical characteristics of total population and according to blood lactate level.

Clinical characteristics	Total (n = 129)	Blood lactate level (mmol/L)			*P* value
		Mild (1–2.5) (n = 60)	Mod. (2.5–4) (n = 46)	Severe (≥ 4) (n = 23)	
Blood lactate (mmol/L)	3.3 ± 2.3	2.0 ± 0.3	3.0 ± 0.4^§^	7.4 ± 2.5^⁋^	**< 0.001**
Age	57.9 ± 31.4	56.2 ± 18.1	59.6 ± 15.7	60.0 ± 15.7	0.424
Female gender	52 (40%)	28 (47%)	17 (37%)	7 (30%)	0.341
**ECOG performance score**					**0.002**
0–1	71 (55%)	43 (72%)	19 (41%)	9 (39%)	
2–4	58 (45%)	17 (28%)	27 (59%)	14 (61%)	
**Ann Arbor stage**					**0.003**
I–III	26 (20%)	20 (33%)	6 (13%)	0 (0%)	
IV	103 (80%)	40 (67%)	40 (87%)	23 (100%)	
Liver involvement present	31 (24%)	8 (13%)	11 (24%)	12 (52%)	0.001
**IPI risk score**					**< 0.001**
Low/intermediate (0–3)	68 (53%)	43 (72%)	15 (33%)	10 (43%)	
High (4–5)	61 (47%)	17 (28%)	31 (67%)	13 (57%)	
Blood LDH (U/L)	2035 ± 2411	2070 ± 2784	2250 ± 2332	1518 ± 1425	0.169
Diabetes mellitus	22 (17%)	7 (12%)	10 (22%)	5 (22%)	0.368
Cognitive/behavioral sx	8 (6%)	0 (0%)	2 (1%)	6 (5%)	**< 0.001**
Blood glucose (mg/dL)	110.3 ± 29.1	112.2 ± 32.2	110.9 ± 29.7	105.2 ± 17.2	0.473
Lymphoma TLG	10,656 ± 9660	7580 ± 7501	11,893 ± 10,561*	16,204 ± 10,137^§^	**< 0.001**

Significant values are in bold.

Mod, moderate; ECOG**,** Eastern Cooperative Oncology Group; FDG, ^18^F-fluorodeoxyglucose; SUVave, average standard uptake value; IPI, International Prognostic Index; LDH, lactate dehydrogenase; sx, symptom; TLG, total lesion glycolysis. Data are represented as means ± standard deviations.

^⁋^*P* < 0.001 compared to all other groups; ^§^*P* < 0.001 compared to the mild group; *P < 0.05 compared to the mild group.

Blood lactate level in the entire study population was 3.3 ± 2.3 mmol/L. Normal blood lactate level is up to approximately 1.0 mmol/L^19^. Severe hyperlactatemia is generally defined as blood lactate > 4.0 mmoL/L^20,21^. Indeed, blood lactate concentration > 4 mmol/L predicts the need for hospital admission for patients in the emergency department^19^. Although, there is no widely accepted criterion for moderate hyperlactatemia, Previous studies have defined this as blood lactate between 2.2 to 4.0 mmol/L^20^ and 2.0 to 4.0 mmol/L^21^. In our study, blood lactate was categorized as mild elevation (1.0–2.5 mmol/L) in 60 cases, moderate elevation (2.5–4.0 mmol/L) in 46 cases, and severe elevation (≥ 4.0 mmol/L) in 23 cases.

Comparison of clinical characteristics between blood lactate groups (Table 1) demonstrated that higher lactate elevation groups had a greater Ann Arbor stage (*P* = 0.003), ECOG score (*P* = 0.002), and International Prognostic Index (IPI) risk score (*P* < 0.001).

As for lymphoma subtypes that may involve the blood, there were seven intravascular DLBCLs, one aggressive NK cell leukemia/lymphoma, two Burkitt lymphomas, three follicular lymphomas, and three Mantle cell lymphomas. However, blood lactate in these 16 subjects remained in the range of the moderate lactate group (2.98 + 1.91). This indicates that risk of blood involvement was not likely a significant contributor to hyperlactatemia severity in our study.

### Associations of blood lactate concentration with lymphoma status and tumor FDG uptake

**Figure 1:**
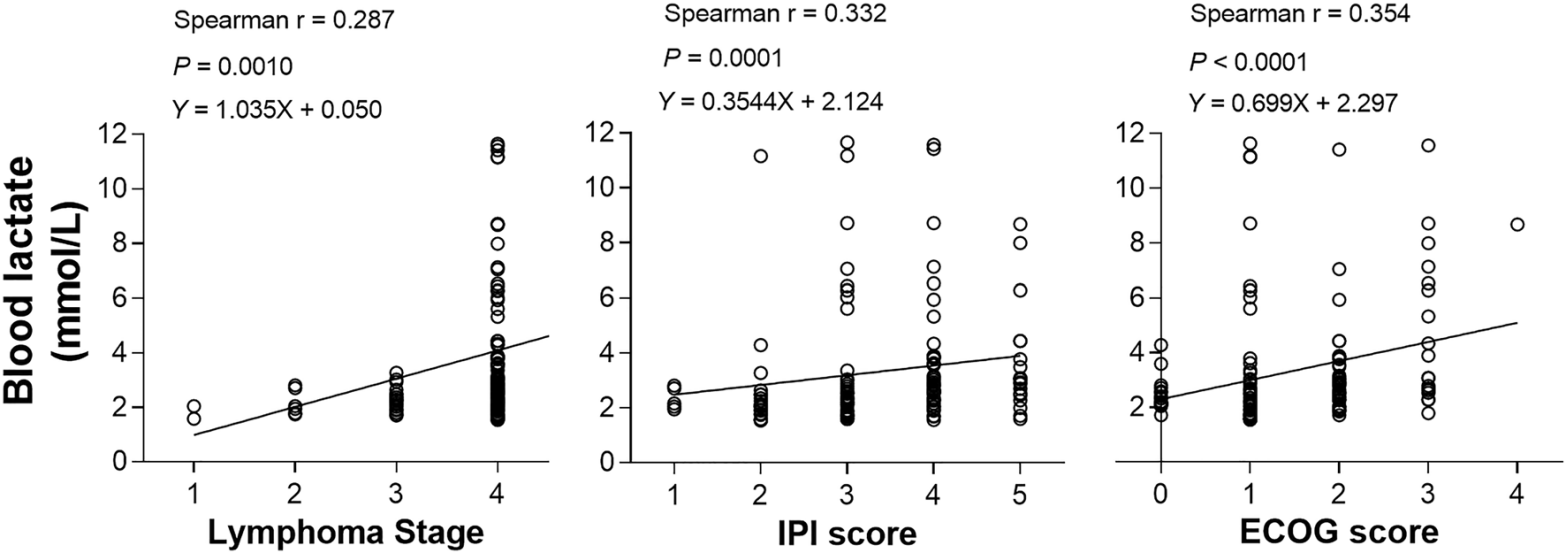
Relationship between blood lactate level and lymphoma status in 129 patients. Blood lactate concentration is elevated in a manner associated with higher lymphoma stage (left) and weakly associated with greater International Prognostic Index (IPI) risk score (middle) and worse Eastern Cooperative Oncology Group (ECOG) performance score (right).

Plotting of blood lactate concentration against lymphoma status revealed a close positive association with higher stage (*P* = 0.0001; Fig. 1). Indeed, all patients with a blood lactate level exceeding 4.0 mmol/L had stage IV disease. Blood lactate also showed a weak but significant association with worse ECOG performance (*P* < 0.0001) and greater IPI risk score (*P* = 0.0001; Fig. 1).

On FDG PET/CT analysis, the lymphoma lesions in our subjects showed a wide range of total lesion glycolysis (TLG), with a mean value of 10,656 ± 9660. Comparison between blood lactate groups demonstrated that severe and moderate elevation groups had high TLG (16,204 ± 10,137 and 11,893 ± 10,561, respectively) that were greater than that of the mild elevation group (7580 ± 7501; *P* < 0.001 and < 0.05, respectively; Table 1).

**Figure 2:**
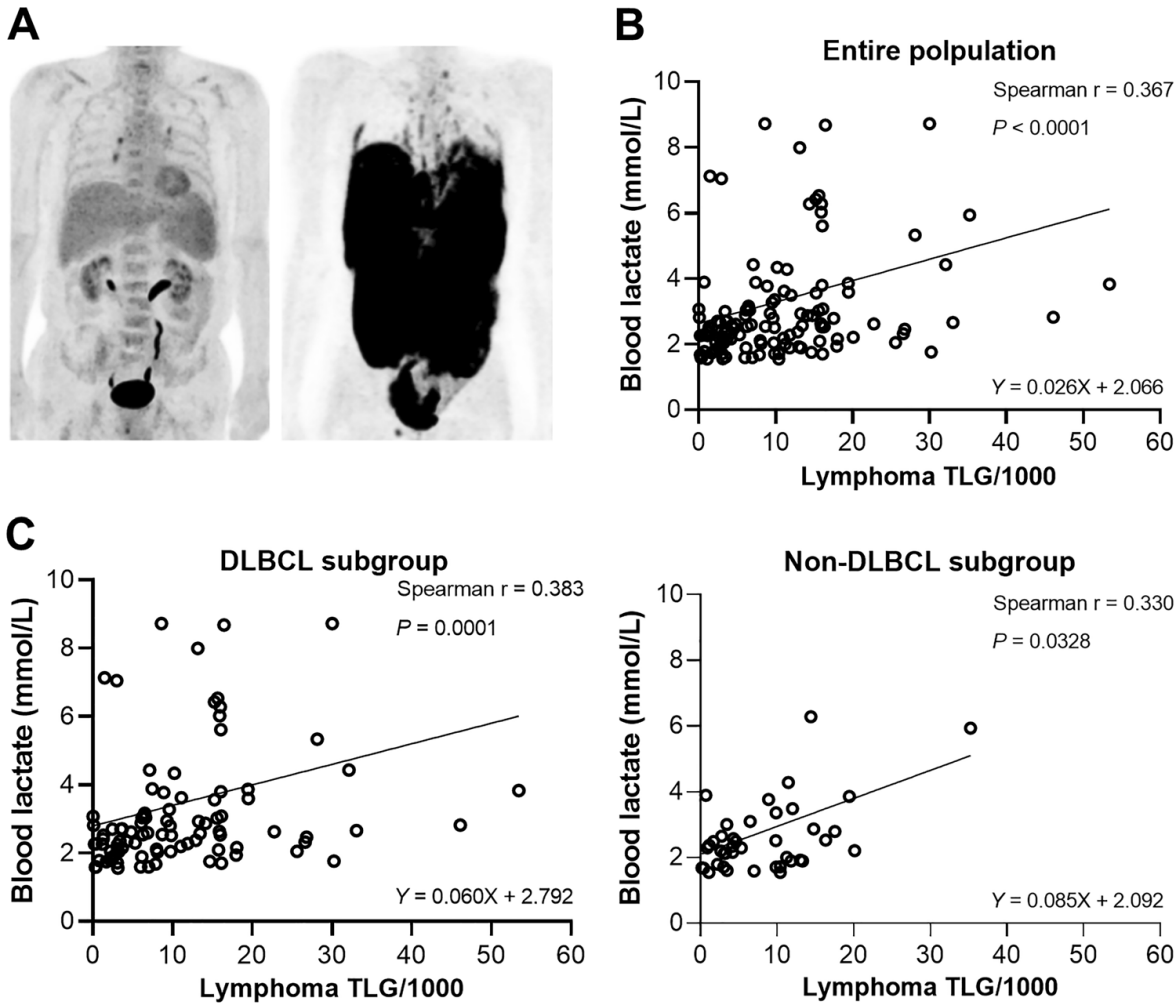
Blood lactate level is correlated with total lesion glycolysis (TLG) measured by FDG positron emission tomography (PET). (**a**) Representative maximum intensity projection FDG torso PET images of stage IV DLBCL patients. The 73-year-old male on the left had mildly elevated blood lactate to 1.6 mmol/L and a low TLG of 379, whereas the 41-year-old male on the right had a high blood lactate of 11.2 mmol/L and high TLG of 38,977. (**b**) Correlation in the entire population between blood lactate concentration and lymphoma TLG with linear regression fitting. (**c**) Correlation between blood lactate concentration and lymphoma TLG for DLBCL (left) and non-DLBCL subgroups.

Figure 2a illustrates maximum intensity projection PET images of a patient with low TLG (379) who had blood lactate mildly elevated to 1.6 mmol/L and another patient with high TLG (38,977) who had blood lactate severely elevated to 11.2 mmol/L.

Plotting the relationship between lymphoma TLG and blood lactate concentration in the entire population demonstrated a significant positive association (Spearman’s r = 0.367; *P* < 0.0001; Fig. 2b). Because the magnitude of correlation may be influenced by lymphoma subtype, we repeated the analysis after dividing subjects into DLBCL (n = 87; Spearman’s r = 0.344; *P* = 0.0011) and non-DLBCL groups (n = 42; Spearman’s r = 0.330; *P* = 0.0328). The results demonstrated similar moderate correlations between lactate and TLG for the two groups (Fig. 2c).

### Brain FDG uptake and associations with lymphoma status

**Figure 3:**
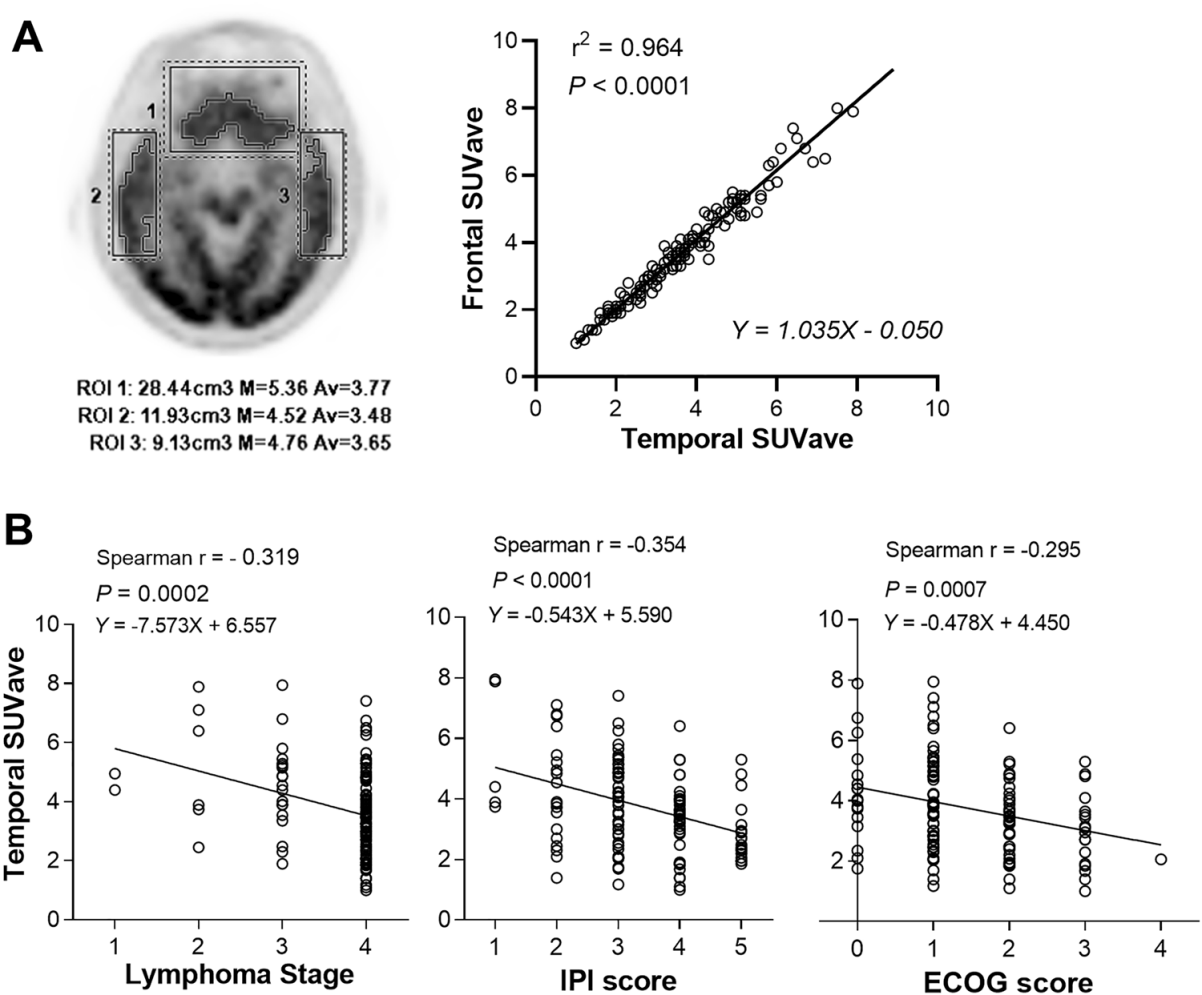
Relationship between brain FDG uptake and lymphoma status. (**a**) Illustration of volumes of interest (VOIs) placed on frontal and temporal lobes (left), and excellent concordance between frontal and temporal cortex uptake levels (right). Cortical boundaries were defined as 40% of the maximum activity. (**b**) Relations between temporal cortex FDG uptake with lymphoma stage (left), IPI risk score (middle), and ECOG performance (right). SUVave, average standard uptake value; ECOG, Eastern Cooperative Oncology Group; IPI, International Prognostic Index.

When we performed region-of-interest analysis of the frontal and temporal cortex average standard uptake value (SUVave) as indices of brain FDG uptake, the two displayed excellent concordance (r^2^ = 0.964; *P* < 0.0001; Fig. 3a). The temporal cortex SUVave was selected for further analysis and showed an average value of 3.7 ± 1.5 for the entire population. Based on this index, 81 subjects (62.8%) had low brain uptake (SUVave < 4.0), while 48 (37.2%) had normal brain uptake (SUVave ≥ 4.0). Subjects with low brain FDG uptake were significantly older (*P* = 0.005), had greater Ann Arbor stage (*P* = 0.002) and IPI risk (*P* < 0.001), and worse ECOG score (*P* = 0.002; Table 2). Those with low brain uptake also had significantly higher blood lactate concentration (*P* < 0.001; Table 2).

**Table 2:** Clinical characteristics according to brain FDG uptake.

Clinical characteristics	Brain FDG (SUVave)		*P* value
	Normal (≥ 4) (n = 48)	Low (< 4) (n = 81)	
Temporal cortex SUVave	5.36 ± 1.24	2.76 ± 0.80	**< 0.001**
Age	53.1 ± 19.1	61.4 ± 14.4	**0.005**
Female gender	23 (48%)	29 (36%)	0.177
**ECOG performance score**			**0.002**
0–1	35 (73%)	36 (44%)	
2–4	13 (27%)	45 (56%)	
**Ann Arbor stage**			**0.002**
I–III	16 (35%)	10 (12%)	
IV	32 (65%)	71 (88%)	
Liver involvement present	7 (15%)	24 (30%)	0.054
**IPI score-based risk**			**< 0.001**
Low to intermediate (0–3)	35 (73%)	33 (41%)	
High (4–5)	13 (27%)	48 (59%)	
Diabetes mellitus	3 (6%)	19 (24%)	**0.012**
Blood glucose (mg/dL)	108.2 ± 29.0	110.7 ± 30.1	0.544
Blood lactate (mmol/L)	2.32 ± 0.96	3.94 ± 2.59	**< 0.001**
Blood LDH (U/L)	1653 ± 1368	2246 ± 2814	0.091

Significant values are in bold.

FDG, ^18^F-fluorodeoxyglucose; SUVave, average standard uptake value; ECOG**,** Eastern Cooperative Oncology Group; IPI, International Prognostic Index; LDH, lactate dehydrogenase. Data are represented as means ± standard deviations.

Plotting temporal cortex SUVave against lymphoma status (Fig. 3b) demonstrated significant inverse correlations with disease stage (Spearman’s r = − 0.319; *P* = 0.0002), IPI risk score (Spearman’s r = − 0.354; *P* < 0.0001), and ECOG score (Spearman’s r = − 0.295; *P* = 0.0007). There was also a significant inverse correlation between temporal SUVave and blood glucose (*P* < 0.0001).

### Associations of brain FDG uptake level with clinical variables

**Figure 4:**
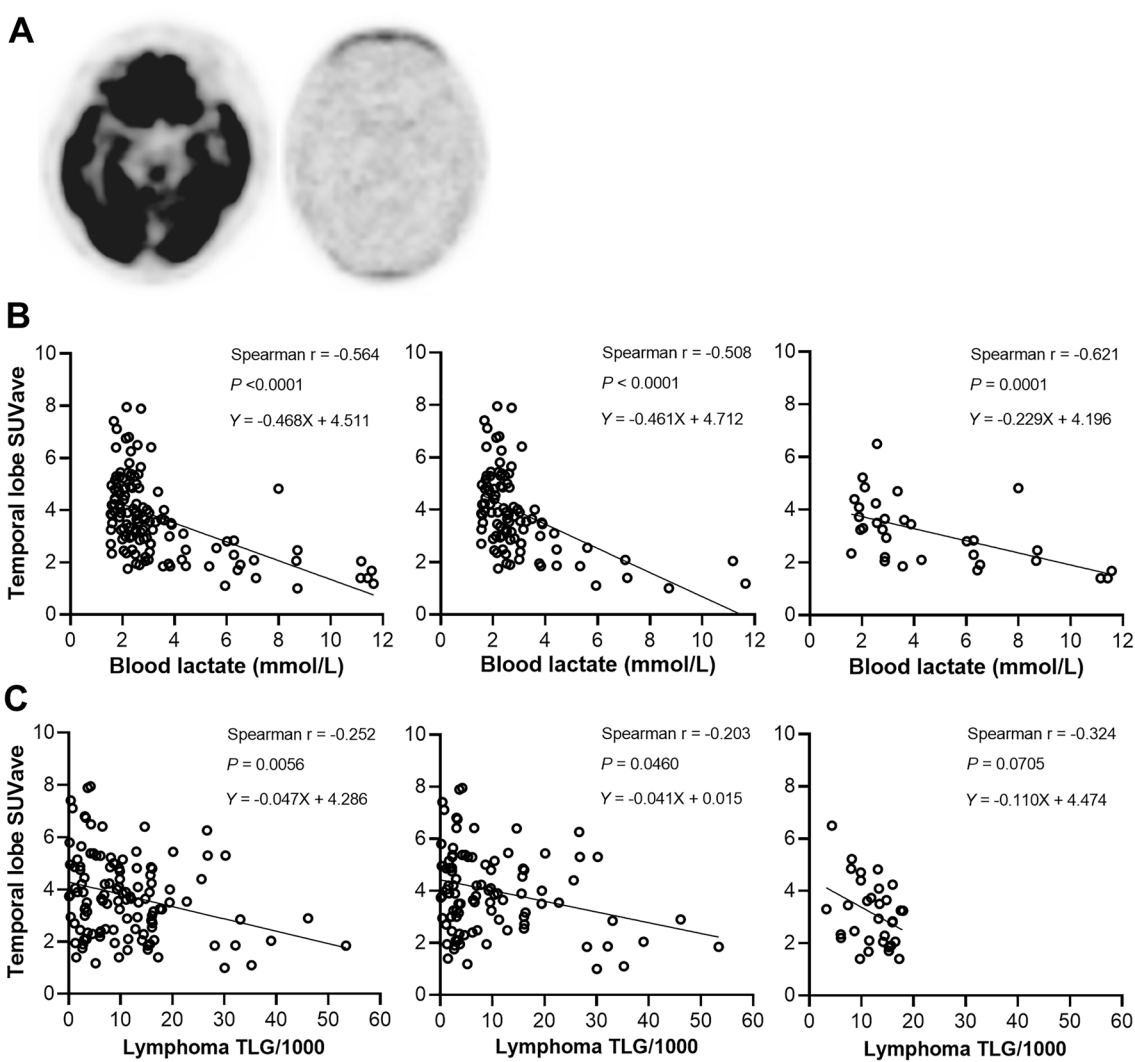
Brain FDG uptake is reduced in a fashion correlating to blood lactate and lymphoma TLG level. (**a**) Representative transaxial FDG brain PET images of a 75-year-old female DLBCL patient who had mildly elevated blood lactate to 1.8 mmol/L and a high temporal cortex SUVave of 6.4 (left) and of a 57-year-old female DLBCL patient with a high blood lactate of 11.7 mmol/L and a severely reduced temporal cortex SUVave of 1.2 (right). (**b**,**c**) Linear regressions demonstrate significant correlations of temporal cortex SUVave with blood lactate (**b**) and lymphoma TLG (**c**). Groups are the entire population (left), the no hepatic lymphoma group (middle), and the hepatic lymphoma group (right).

Figure 4a illustrates PET images of a patient with mild elevated blood lactate and normal brain FDG uptake, and another with severe elevated blood lactate and markedly reduced brain FDG uptake. Plotting the relationship between temporal SUVave and blood lactate concentration revealed a significant inverse correlation (Spearman’s r = − 0.564; *P* < 0.0001; Fig. 4b). Temporal SUVave further showed a significant inverse correlation with TLG level (Spearman’s r = − 0.252; *P* = 0.0056; Fig. 4c).

Since hepatic metabolism accounts for 90% of lactate clearance^32^, we assessed whether impairment of liver function might have contributed to increase lactate accumulation as previous reported^7^. Our subjects were thus categorized according to presence of lymphoma involvement in the liver and/or severe liver function (LFT) abnormalities. The former was based on PET and enhanced AP CT findings, and the latter was defined by the presence of any of the followings at the time of PET: blood SGOT or SGPT > 160 U/L, alkaline phosphatase (ALP) > 600 U/L, or total bilirubin > 6.0 mg/dL (all are four-times that of upper normal range). Among those without hepatic lymphoma (n = 97), there was no difference in blood lactate (2.80 ± 0.88 vs. 2.92 ± 1.82), TLG (5760 ± 6392 vs. 10,637 ± 11,157), and temporal SUVmax (3.46 ± 0.64 vs. 3.99 ± 1.73) between those with (n = 9) and without severe LFT abnormality (n = 88). Among those with hepatic lymphoma (n = 32), there was also no difference in blood lactate (3.83 ± 1.94 vs. 5.57 ± 3.78), TLG (11,886 ± 4144 vs. 12,334 ± 3904), and temporal SUVmax (3.22 ± 1.13 vs 3.05 ± 1.38) between those with (n = 18) and without severe LFT abnormality (n = 14). However, those with hepatic lymphoma had significantly greater blood lactate (4.59 ± 3.05 vs. 2.91 ± 1.75; *P* = 0.0002) and lower temporal SUVmax (3.14 ± 1.25 vs. 3.94 ± 1.66; *P* = 0.0137) compared to the those without hepatic lymphoma.

Given these results, we repeated correlation analysis in patients sub-grouped according to presence of liver involvement. The results showed that the inverse correlation between temporal SUVave and blood lactate remained strong in both ‘no hepatic lymphoma’ (Spearman’s r = − 0.508; *P* < 0.0001) and ‘hepatic lymphoma’ groups (Spearman’s r = − 0.621; *P* = 0.0001; Fig. 4b). The inverse correlation between temporal SUVave and TLG remained weak in the ‘no hepatic lymphoma’ group (Spearman’s r = − 0.203; *P* = 0.0460), but significance of difference was lost in the ‘hepatic lymphoma’ group (Spearman’s r = − 0.324; *P* = 0.0705; Fig. 4c).

We also checked whether any of our subjects had symptoms of cognitive or behavioral change at the time of PET. There was one case of confused mentality who was diagnosed with tumor lysis syndrome and died two days after the PET study. There were also cases with dizziness (n = 1), agitation that required sedation (n = 1), drowsiness (n = 4), and delirium (n = 1). Notably, all 8 of these subjects showed low temporal SUVmax (< 4.0). Six and two cases had severely and moderately increased blood lactate, respectively, Table 1).

### Univariable and multivariable explanatory variables for brain FDG uptake level

To dissect the explanatory variables that determine brain FDG uptake in our patients, univariable linear regression analysis was performed. The results demonstrated that ECOG score (*P* = 0.001), IPI risk score (*P* < 0.001), lymphoma stage (*P* = 0.001), blood glucose (*P* < 0.001), blood lactate (*P* < 0.001), and TLG (*P* < 0.001) were all inversely correlated with log temporal cortex SUVave (Table 3).

**Table 3:** Clinical and PET predictors of log temporal SUVave.

Univariable predictors	Pearson r		*P*	
Blood lactate	− 0.643		**< 0.001**	
Blood glucose	− 0.389		**< 0.001**	
Lymphoma TLG	− 0.286		**0.001**	
IPI risk score	− 0.326		**< 0.001**	
ECOG performance score	− 0.289		**0.001**	
Ann Arbor stage	− 0.289		**0.001**	
Blood LDH level	− 0.135		0.144	

Multivariable predictors	B	SE	β	*P*
Blood lactate	− 0.039	0.006	− 0.444	**< 0.001**
Blood glucose	− 0.003	0.000	− 0.411	**< 0.001**
Log TLG	− 0.005	0.001	− 0.288	**< 0.001**

Significant values are in bold.

ECOG, Eastern Cooperative Oncology Group; IPI, International Prognostic Index; LDH, lactate dehydrogenase; TLG, total lesion glycolysis; S.E., standard error.

Finally, multivariable linear regression analysis performed with significant univariable correlates revealed that blood lactate (B, − 0.039; *P* < 0.001), blood glucose (B, − 0.003; *P* < 0.001), and TLG (B, − 0.005; *P* = 0.001) were significant independent explanatory variables for log temporal cortex SUVave.

## Discussion

Our study of non-Hodgkin’s lymphoma patients who underwent blood tests for suspected lactate elevation demonstrated the subjects to have a mean blood lactate concentration of 3.3 mmol/L. This is quite similar to the mean blood lactate of 3.25 mmol/L observed in a recent study of hospitalized lymphoma patients who turned out to have progressive disease^22^. The study further found lactate concentrations exceeding 5.0 mmol/L in 25% of the patients^22^. In our study, 35.7% of subjects had lactate concentrations of 2.5–4 mmol/L, and 17.8% of subjects had lactate concentrations exceeding 4.0 mmol/L. The latter level has been used as a definition for severe hyperlactatemia^23^.

Elevated serum lactate, which is most often associated with hematological malignancies^1,7,10,14^ and less often with solid tumors^7,24–28^, is generally attributed to a metabolic cancer hallmark called the Warburg effect. This reprograming of tumor metabolism is characterized by the consumption of huge amounts of glucose for heightened glycolytic flux that results in increased lactate release into the circulation^29^. The PET/CT images of our subjects did not show evidence of necrotic tissue in the lymphoma lesions, which is consistent the general radiologic feature of predominantly homogenous nonnecrotic tumor. This supports the notion that our study patients had type B hyperlactatemia unassociated with tissue hypoxia.

Previously, higher blood lactate levels were observed in glioma patients with greater tumor grade^26,28^, brain tumor patients with greater proliferative Ki-67 index^28^, and colorectal cancer patients with metastatic disease^7,25^. In our study, the higher blood lactate groups had greater tumor TLG level, a volume-based metabolic parameter of tumor burden that is increasingly used in the clinics^30^. This indicates that blood lactate elevation in our subjects was likely caused by a large burden of tumors with heightened glycolysis. Indeed, a comparison between blood lactate concentration and tumor TLG level revealed a significant positive correlation. Hence, this is the first demonstration that blood lactate in lymphoma patients is elevated in a manner proportional to glycolytic tumor burden.

Notably, PET images demonstrated low brain FDG uptake in a substantial 62.8% of our study subjects. This group of patients had greater Ann Arbor stage and IPI risk score, worse ECOG performance, and higher blood lactate level. Since glycolytic tumors consume large amounts of glucose^30^, one might attribute lower brain FDG uptake to diversion of glucose away from the brain toward the glycolytic tumor. However, this is unlikely because these subjects did not show low blood glucose. Furthermore, their PET images displayed normal FDG activity in blood pool and major organs, indicating lack of glucose deficiency. Rather, the reduced cortical FDG uptake we observed appears to represent the preferential use by the brain of lactate that has become available at greater amounts. Indeed, subjects with low brain FDG uptake had significantly elevated circulating levels of lactate, which is now recognized to serve as an alternate fuel for the human brain^18^. When delivered in sufficient amounts, lactate can replace glucose as the brain’s energy source, even under euglycemic conditions^31,32^. Taken together, our findings indicate that increased release of lactate by glycolytic lymphoma tissue is preferentially used by the brain, which leads to glucose sparing and reduced brain FDG uptake on PET images.

Importantly, we were able to establish a linear relationship between degree of suppression in brain FDG uptake and magnitude of blood lactate elevation. Unlike cerebral glucose uptake that is constant regardless of blood level, cerebral lactate uptake is arterial concentration-dependent^33,34^. Therefore, glucose sparing (and thus reduced FDG uptake) in the brain occurs in a manner proportional to increased levels of blood lactate, which explains the linear relationship we observed. This implies that considerable degrees of brain FDG reduction on PET studies of lymphoma patients could be used as a sign of clinically significant hyperlactatemia that requires attention and prompt testing.

According to Cohen’s conventions, Spearman’s r values of 0.3 to 0.5 are considered moderate and those exceeding 0.5 are considered strong correlations (ref^35^). We thus observed a strong correlation between lactate and temporal SUV and a moderate correlation between lactate and TLG. The weak correlations that were found between some variables appear likely due to other factors also contributing to the observed measurements.

We did not observe differences in blood lactate, TLG, or brain FDG uptake according to the presence of severe LFT abnormalities. Hence, blood SGOT, SGPT, ALP, or bilirubin levels do not appear to necessarily correlate with the liver’s ability to metabolize circulating lactate. On the other hand, the presence of hepatic lymphoma led to significantly greater increases of blood lactate and reductions of temporal SUVmax. Yet, although temporal SUVave and TLG no longer showed significant correlation in patients with hepatic lymphoma, the inverse correlation between blood lactate and temporal SUVave remained strong regardless of whether hepatic lymphoma was present. These findings suggest that although lymphoma involvement in the liver might contribute to decreased lactate clearance, an inverse relation between blood lactate and brain FDG uptake is maintained.

Kidney dysfunction can also lead to lactate accumulation^36^. However, subjects with increased serum creatinine (> 1.2 mg/dL; n = 19) did not show significantly different blood lactate (3.55 ± 1.90 vs. 3.29 ± 2.31 mmol/L) or temporal SUVmax (3.0 ± 1.0 vs. 3.9 ± 1.7) compared to those with normal serum creatinine, indicating that renal function was not a significant contributor to our findings.

In addition to serving as a marker of severe illness, the clinical relevance of elevated blood lactate includes its role as energy source^37^, paracrine or endocrine-like effects^34^, and potential influence on neuronal function^38,39^. In our study, all 8 cases who showed neurologic symptoms displayed low temporal FDG uptake and elevated blood lactate (> 6 mmol/L in six and > 11 mmol/L in two cases). This suggests that in certain situations, high blood lactate with low brain FDG uptake may be associated with risk of neurologic symptoms, although further studies will be needed to clarify this possibility.

Our findings raise the possibility that increased blood lactate and lowered brain FDG uptake may offer prognostic value. However, our study subjects consisted of 13 different lymphoma subtypes (with highly variable prognosis) to investigate the relation between hyperlactatemia and brain FDG uptake. Even the predominant DLBCL subtype included 59 patients at initial diagnosis and 20 at disease relapse. There were also differences in treatment regimen. Moreover, the wide range of Ann Abor stage, ECOG performance, and IPI risk further contributed to subject heterogeneity that made prognostic analysis difficult. The inability to analyze the impact of brain FDG uptake and blood lactate on patient outcome due to severe subject heterogeneity is a limitation of our study. Future investigations on a more homogeneous population of lymphoma patients are thus warranted to clarify the prognostic values of lowered brain FDG uptake and increased blood lactate.

## Conclusion

In lymphoma patients, blood lactate elevation occurs as a result of glycolytic tumor burden as measured by TLG on FDG PET. In these cases, brain FDG uptake is decreased in a manner inversely correlated to blood lactate concentration, indicating glucose sparing by lactate as an alternative fuel. FDG PET/CT can therefore identify lymphoma patients with high glycolytic tumor burden at risk of hyperlactatemia and may provide estimates of its severity by visualizing the degree of reduction in brain FDG uptake.

## Materials and methods

### Patient population

This retrospective study was approved by the Samsung Medical Center Institutional Review Board with exemption for informed consent, and all methods were performed in accordance with the relevant guidelines and regulations. Study subjects were selected from consecutive patients with histology-confirmed non-Hodgkin’s lymphoma at initial staging or re-staging between November 2008 and April 2018 at our institution, who underwent blood lactate tests and FDG PET/CT imaging on the same day. Patients with lymphoma in the brain were excluded. Finally, a total of 129 patients were included for analysis. The most common lymphoma subtype was diffuse large B cell lymphoma (n = 87, 67.4%); the remaining subtypes were T cell in 21, NK/T cell in 11, follicular in 3, mantle cell in 3, marginal zone in 2, and Burkitt lymphoma in 2 patients. Although the subjects were consecutive lymphoma patients, they were selected for those who had blood lactate measurements on the same day of PET/CT for suspected hyperlactatemia. The population was thus biased for lymphoma subtypes associated with high lactate production, and do not reflect subtype frequency.

### Medical record review

Clinical information obtained from the institutional information system included age, sex, ECOG performance score, B symptoms, Ann Arbor stage, and presence of liver involvement. Blood glucose and lactate concentrations were measured from venous blood sampled on the same day as the PET/CT. Other baseline laboratory data acquired within one week of PET/CT included serum LDH, hemoglobin level, white blood cell count, and platelet count. The IPI risk score was calculated from these data.

### FDG PET/CT imaging

Patients fasted for at least 6 h, and blood glucose was < 200 mg/dl at the time of FDG injection. Imaging was performed 60 min after injection of 5 MBq/kg FDG on a Discovery LS (GE Healthcare) or an STe PET/CT scanner (GE Healthcare) without intravenous or oral contrast. Whole-body CT was performed with a continuous spiral technique with an 8-slice helical CT (140 keV; 40–120 mA; section width, 5 mm; Discovery LS) or a continuous spiral technique with a 16-slice helical CT (140 keV; 30–170 mA; 3.75 mm; STe). After CT, an emission PET scan was obtained from head to thigh. This was collected either at 4 min per frame in 2-D mode with attenuation-corrected images (4.3 × 4.3 × 3.9 mm) and reconstructed using an ordered-subset expectation maximization algorithm (28 subsets, 2 iterations; Discovery LS) or at 2.5 min per frame in 3-D mode with attenuation-corrected images (3.9 × 3.9 × 3.3 mm) and reconstructed using a 3-D ordered-subset expectation maximization algorithm (20 subsets, 2 iterations; Discovery STe).

### PET/CT analyses for glycolytic tumor burden and brain FDG uptake

FDG PET/CT images were analyzed by two experienced nuclear medicine physicians on a dedicated GE Advantage workstation 4.4 (GE Healthcare). From transaxial PET tomographs of the torso, a SUV threshold-based isocontour method was used to depict the boundaries of each lymphoma lesion. The volumes of interest (VOIs) were automatically drawn around each lymphoma lesion by applying a 41% threshold of the local SUVmax. Volumes were manually edited to remove physiological activity including that of the brain, heart, liver, kidney, and bladder. Mild diffuse bone marrow uptake consistent with reactive hyperplasia was not included. The metabolic tumor volume (MTV) was the summed volume (ml) of voxels with FDG uptake exceeding the threshold. TLG was the sum of the product of MTV and average SUV (SUVave) of all lesions. Our analyses focused on TLG that represents the total quantity of glycolytic tumor tissue rather than MTV that is simply its volume.

Brain FDG uptake was measured from transaxial PET tomographs with the orbitomeatal line as a reference. Volumes of interest (VOIs) were manually placed over bilateral frontal and temporal lobes, and boundaries of cerebral cortices were defined as 40% of maximum activity (Fig. 3a). From these VOIs, SUVave of the frontal and temporal cortices was measured.

### Statistical analyses

Continuous variables are described as means and standard deviations (SD). The normality of continuous clinical data was tested using the Kolmogorov–Smirnov test, which showed that they did not follow normal distribution. Hence, age, glucose, lactate, LDH, and TLG were analyzed by nonparametric Mann–Whitney tests to compare the two brain FDG uptake groups and by nonparametric Kruskal–Wallis tests to compare the three blood lactate groups. The latter was followed by multiple pairwise comparisons with significance values adjusted by Bonferroni correction for multiple tests. The categorical and discrete data were compared by Pearson’s chi-square tests. Associations of blood lactate concentration or temporal cortex SUVave with clinical variables were analyzed by nonparametric Spearman’s analysis with rank correlation coefficient (r) measurements. Univariable and multivariable regression analysis was performed using SPSS software version 17.0 (SPSS, Chicago, IL). We applied a stepwise multivariable linear regression model with changes in temporal cortex SUVave as a dependent variable and age, stage, lactate, glucose, TLG, ECOG score, IPI score, and LDH level as independent variables. A *P* value of less than 0.05 was accepted as the threshold for inclusion.

### Ethical approval

This study was approved by the Samsung Medical Center Institutional Review Board.

## Data Availability

The datasets generated and analyzed during the current study are available from the corresponding author on reasonable request. (The data are not public because they contain personal information that was provided for research purpose only).
